# The potential and challenges of an online Bible study group for persons with disabilities

**DOI:** 10.4102/ajod.v14i0.1721

**Published:** 2025-09-06

**Authors:** Talia Opperman, Michelle Botha

**Affiliations:** 1 Division of Disability and Rehabilitation Studies, Department of Global Health, Faculty of Medicine and Health Sciences, Stellenbosch University, Cape Town, South Africa

**Keywords:** disability, spirituality, digital access, well-being, inclusion, Christianity, fellowship, support

## Abstract

**Background:**

Research shows that persons with disabilities can derive significant benefits from participating in religious communities and activities. However, they often face significant physical and attitudinal barriers to participation. The use of digital platforms for religious gatherings has increased since the coronavirus disease 2019 (COVID-19) pandemic and may hold potential to promote accessible religious participation.

**Objectives:**

This study explored the benefits and challenges of an online Bible study group for persons with disabilities hosted within a Christian Pentecostal congregation in Cape Town, South Africa.

**Method:**

A phenomenological study design was adopted, and data were gathered from four participants using semi-structured interviews. Thematic analysis was employed.

**Results:**

Although some components of the online environment were found to be beneficial for participants’ spiritual well-being, other components were shown to be challenging, such as technological barriers, a lack of direct physical interaction and feelings of isolation. The digital space provided only partial inclusion and does not represent a full solution to exclusionary religious spaces.

**Conclusion:**

Church leadership must engage more rigorously with strategies of disability inclusion, which may include digital components.

**Contribution:**

This research contributes to the fields of disability studies, religious inclusion and digital engagement. Recommendations include the use of adaptable technologies, leadership training in disability inclusion within a religious space and developing more engaging platforms to foster inclusion within these religious spaces.

## Introduction

Scholars have pointed out that, despite a theological focus and moral imperative towards inclusivity in Christianity, there is a lack of strategies to effectively include persons with disabilities in church and other religious gatherings (Carter et al. 2023; Retief 2016). Hardwick (2021) offers insight into church planning, asserting that the needs of persons with disabilities are frequently regarded as secondary issues, handled only after the fulfilment of the church’s perceived fundamental purposes of preaching and outreach. This lack of inclusion is unfortunate as research by Lorenzo and Duncan (2020) and Carter (2024) highlights the potential benefits of spiritual well-being derived from religious practices for persons with disabilities, including promoting coping, social inclusion and better quality of life. Through spiritual frameworks, persons with disabilities receive profound sources of belonging, identity and meaning (Imhoff 2017). However, when religious communities do not make intentional efforts to include persons with disabilities, they may face physical barriers (such as inaccessible buildings) and attitudinal barriers (rooted in stigma related to religious discourse) to participating in church.

Regarding the latter, the moral or religious model of disability, as seen in several faiths including Christianity, has been associated with the potential for discriminatory practices, as it claims that disability is a result of a moral failing on the part of an individual, family or community (Andrews 2016). Further, Christian beliefs have been criticised for prioritising the healing of disabled bodies, which supports a normalising drive rather than the acceptance of diversity in the church (Botha in press; Imhoff 2017). While some religious interpretations may associate disability with sin, a contrary belief holds that each person is made in the likeness of God and has inherent value and divine purpose regardless of ability (Yong 2007). For instance, Isaac, who is blind, and Moses, who stutters, are protagonists in sacred texts, showing that individuals with disabilities are not just subjects of charity but vital to the holy narrative (Imhoff 2017). Similarly, scholars have challenged limiting perspectives through promoting the idea of an accessible God, who is familiar with and present in the midst of suffering, which is viewed not as punishment but as an opportunity for spiritual growth and fulfilment (Creamer 2012).

Therefore, fostering an inclusive church environment that upholds the rights and dignity of persons with disabilities requires a multi-level approach. The United Nations Convention on the Rights of Persons with Disabilities (UNCRPD) sets forth the objective of complete inclusion for persons with disabilities, which refers not merely to accessible surroundings but also to the acknowledgment of persons with disabilities as equal participants in social and economic spheres (UN 2007). Genuine inclusion, therefore, goes beyond the mere elimination of physical barriers, such as stairs or inaccessible seating; it also requires dismantling social and relational barriers that impede full participation (Thomas 2004). Churches should endeavour to become more inclusive places by reevaluating their policies, values and ways in which persons with disabilities are involved and by actively addressing the physical and attitudinal barriers they experience (Yong 2010; Zondi 2020). It is essential that the perspectives of persons with disabilities are held at the centre of this process.

This study explored a measure to promote disability inclusion in a local church congregation, namely, an online Bible study group. The group was started during the coronavirus disease 2019 (COVID-19) lockdowns by a member of the church congregation (a person with a disability) who had identified a gap in terms of opportunities for persons with disabilities to participate in church activities because of access challenges. The COVID-19 pandemic, which started in March 2020, had a substantial influence on regular church attendance while also causing one in five individuals throughout the world to become housebound (Bryson, Andres & Davies 2020). A hurdle was faced by many believers because of the closing of physical church facilities and the cessation of in-person services. While societies faced the limiting of their movement through lockdown measures, some persons with disabilities drew attention to the fact that such limitations have been and remain part of their daily reality (Lourens & Watermeyer 2023). This was the case for the participants in this study.

Despite its challenges, COVID-19 provided an opportunity as persons began to explore what remote platforms could offer in terms of maintaining participation in spiritual activities (Bryson et al. 2020). However, considerations on the potential and operation of the ‘online church’ far pre-date the pandemic, as seen in Hutchings (2011). Hutchings (2011) states that online churches can serve as digital equivalents to physical congregations, providing opportunities for worship, fellowship and support. Virtual space provides a distinct opportunity for establishing connections and experiencing a sense of belonging. Hutchings (2011) examines the substantial transformations in religious engagement that the development and increasing use of online platforms has brought about. In addition to making it easier for religious teachings to be conveyed, these platforms help to build communities around common values and beliefs. Others consider that the sick, aged and individuals with disabilities may be isolated from spiritual connection and have accepted that the online church may be credible and helpful to their ability to share a fulfilling religious experience (Russell 2016).

However, while virtual attendance may help persons stay connected to their faith, it cannot replace the physical experiences that come with worshiping in-person. According to the research, traditional religious practices, which often include group ceremonies and physical contact, are important for mental health and social support (Upenieks et al. 2023). In addition, a lot of religious activities stress fellowship, which is usually thought to be better when people are physically together (Hutchings 2011). While the embracing of remote platforms holds potential for developing the spiritual well-being of persons with disabilities, there are undoubtedly tensions between inclusion and exclusion in the digital realm, especially as lockdown restrictions were lifted and traditional in-person church services resumed.

Understanding the expectations and experiences of individuals with disabilities in the digital religious realm is therefore important to provide valuable insights into the effectiveness of virtual religious practices in meeting the spiritual needs of persons with disabilities. To this end, this study sought to comprehend the experiences of participants in an online Bible study group, including positive and negative experiences, feelings of belonging or exclusion within the online community, and their expectations and intended outcomes with a view to make recommendations for how churches can promote greater inclusion of persons with disabilities both online and offline. The study aims to explore the experiences of persons with disabilities of participation in an online Bible study group hosted by a Pentecostal church in Cape Town, South Africa.

## Research methods and design

### Research design

A phenomenological methodology was employed for this research. Phenomenology, as a qualitative approach, aims to comprehend the fundamental elements of individuals’ lived experiences through focusing on individuals’ interpretations of specific occurrences (Van Manen & Van Manen 2021). Consistent with phenomenological methodology, semi-structured interviews were selected as the preferred method for data collection. This enables the researcher and participants to pursue a topic in a more fluid way than with structured approaches, making it possible to raise and explore emerging insights (Adams 2015). The decision to employ semi-structured interviews was supported by O’Leary and Hunt (2017), who state that the research topic should dictate the selection of methodologies and procedures.

### Research setting

The Bible study group is hosted under the auspices of a Christian Pentecostal church in Cape Town, South Africa. The Pentecostal movement forms part of a broader Christian renewal movement. It places strong emphasis on the presence of the Holy Spirit – understood as God’s spirit who indwells, and operates among, believers in Jesus Christ to provide direction, comfort and discernment (Retief 2016; Yong 2010). Scholars such as Yong (2010) and Sande and Ringson (2021) critically examine the theological stance on disability within the renewal movement, particularly the strong emphasis it places on miraculous healing. It often rests on the presumption that disability is a deficit or a sign of spiritual brokenness requiring divine intervention. Such a focus can inadvertently reinforce an assumption that life with disability holds no spiritual value and, consequently, hinder the inclusion of persons with disabilities who may feel marginalised or pressured to seek healing, contrary to their own desires or beliefs (Sande & Ringson 2021; Yong 2010).

The church includes around 1500 members who meet regularly for church services and other activities in-person. The Bible study group was started by a member of the church, a person with a disability, to address access barriers faced by persons with disabilities to attending in-person church activities, notably related to inaccessible transport and church infrastructure. The Bible study meets online to study the Bible, pray and socialise together. It is also important to state here that the researcher (the first author) is a member of the larger congregation. As someone who has experienced personal growth through weekly attendance of a Bible study group, the researcher was motivated to study an online Bible study for persons with disabilities to understand what role this form of engagement with scripture can play in their lives. Her positionality as an insider researcher will be discussed in more detail below.

### Sampling and recruitment

Total population sampling was used, as advised by O’Leary and Hunt (2017). For small, data-rich groups, total population sampling remains an effective method because it involves including every member of the population who meets the study criteria, thereby maximising the depth and breadth of information collected. For inclusion in this research, participants were required to be over 18 years old, be active members of the Bible study group and self-identify as a person with a disability. Anyone under the age of 18 years or who did not self-identify as having a disability was excluded. After applying these criteria to the total population of the Bible study group, four participants were identified as eligible. Patton (2002) says that information-rich cases are important for getting a deep understanding of what people go through every day. Larger samples are not necessarily required for generating rich descriptions (Palinkas et al. 2015). The final group of four people in this research is thought to be sufficient to obtain useful details about the phenomenon being examined.

The recruitment process began with seeking permission to conduct the study from church leadership. A letter was sent via email describing the project’s aim and objectives. Upon receiving approval, the researcher requested an opportunity to address the group at one of their regular meetings. This session enabled the researcher to introduce the project, talk about the research goals and answer any questions that possible participants might have. The participants were given the contact details of the researcher during this meeting so they could contact her privately if they had any more questions and to consent to participate. Each participant received the project information sheet and consent form before data collection took place.

### Participants

Participants come from different backgrounds and are persons with various disabilities. Table 1 describes the participant demographics. The names provided are pseudonyms, which the participants chose to maintain confidentiality.

**TABLE 1:** Participants’ demographics.

Pseudonym	Year joined	Disability	Reason for joining the group	Key themes in contributions
Peace	2023	Yes Visual impairment	Seeking community and spiritual belonging despite physical limitations.	Belonging, emotional support and the need for deeper connections
Love	2023	Yes Mobility issues	Expressing gratitude to God and remaining active in a faith community	Empathy, emotional support and compassionate actions
Courage	2023	Yes Mobility difficulties	Seeking spiritual support and a family-like community in managing chronic illness	Resilience, mutual support and perseverance
Kindness	2023	Yes Physical accessibility challenges	Advocating for inclusion and acceptance within the faith community	Empathy, inclusion and kindness in community-building

### Data collection

Semi-structured interviews were conducted remotely using voice and video calling via WhatsApp – the same platform used for the Bible study group. This approach was used to guarantee accessibility and inclusivity, considering the many physical and geographical limits the participants identified. An interview guide with semi-structured questions was developed to facilitate the interviews. Interviews took place in English, which is the primary language used in the group, though participants were given the option to conduct the interview in Afrikaans, another predominant language in the church congregation. The length of the interviews ranged from 45 min to 60 min.

The fact that the interviews were semi-structured allowed for flexibility in the conversation while ensuring that the primary themes relating to the research question were maintained (Bryman 2008). Interviews were arranged at convenient times for the participants. Interviews were staggered into multiple shorter sessions where needed, allowing participants to take breaks. Being new to interviewing methods, the researcher prepared by doing a mock interview with the supervisor (the second author) where she received feedback on her approach.

Pseudonyms were allocated to participants during the transcription phase to safeguard their identities. The participants themselves selected these pseudonyms, which added a dimension of personal engagement. The pseudonyms were chosen to reflect positive qualities, with names such as ‘Love’, ‘Peace’, ‘Kindness’ and ‘Courage’ being inspired by the ‘fruit of the Spirit’ from Galatians 5:22–23. The church organisation is also not identified in this research.

Recorded interviews were transcribed and anonymised, and both the digital recordings and physical copies of the transcripts were securely stored. Hard copies of the data and consent forms were stored in a file cabinet secured with a lock. Digital files were password-protected in a folder on the researcher’s laptop and backed up in case of loss or theft. Data will be retained and destroyed after 5 years.

### Data analysis

Inductive thematic analysis was used in this study, meaning that themes were not predetermined (Clarke & Braun 2014). The phenomenological paradigm emphasises the comprehension of participants’ lived experiences, and, to this end, thematic analysis was used to carefully examine the data, looking for common themes or patterns in participants’ answers. This method helps organise detailed and sometimes complicated narratives into clear categories, showing what is similar and different in participants’ experiences.

Interviews were transcribed and the researcher read and re-read the transcripts to familiarise herself with the data (Clarke & Braun 2014). The researcher then began to code the transcripts, involving identifying recurring ideas and experiences in the dataset (Clarke & Braun 2014). Codes were then organised into themes, which involved re-reading transcripts to make sure that the themes accurately caught the main ideas and feelings of the participants. Each theme was given a descriptive title and was supported by direct quotations and instances from the interviews, anchoring the research in the participants’ own expressions (Clarke & Braun 2014).

### Rigour and trustworthiness

Ensuring rigour and trustworthiness is a criterion for promoting quality and minimising biases in qualitative work. Rigorous research must guarantee that the results as closely as possible represent the meanings that participants produce in relation to the phenomenon of interest (Murphy & Yielder 2010). To this end, open-ended research questions facilitated participants to express their thoughts and opinions without any restrictions, thereby yielding comprehensive and elaborate insights into their experiences (Creswell & Poth 2016). Moreover, after conducting the initial analysis and identifying preliminary themes, member checking was conducted with participants via WhatsApp. This was to confirm that participants comprehended and concurred with the interpretations derived from their responses (Shenton 2004). If discrepancies or misunderstandings were identified during member checking, the researcher revisited the data and revised the interpretations to ensure accuracy. Further, this study maintained trustworthiness by regular discussions with a research supervisor, who offered an external perspective that could challenge and refine emerging themes. Debriefing with the supervisor provided an important safeguard against subjectivity, critically questioning potential biases and ensuring the research adhered to ethical and methodological standards.

### Positionality

A researcher’s positionality, including their background, beliefs and experiences, can influence how data are collected and interpreted in qualitative research. The researcher in this study was careful to reflect consistently on her status as an insider to the Pentecostal faith and to this church community. As someone who shared a common faith with the participants, the researcher was able to build rapport and trust through a common language, which facilitated more open and candid responses. However, the researcher was also keenly aware of the potential for bias, particularly when facing critique of the church from the participants. In this regard, discussions with the research supervisor and reflective journalling were useful to unpick conscious bias and to bring unconscious bias to the surface (Denzin & Lincoln 2011).

Journalling was also valuable to ensure the dependability of the research, referring to transparency concerning study design, data collection and analysis procedures. By maintaining detailed field notes in her journal, as well as interview transcriptions and analytical memos, the researcher ensured that each step was transparent and traceable.

### Ethical considerations

The research received ethics approval from the Health Research Ethics Committee of Stellenbosch University (S23/10/258) for the period 11 March 2024 to 10 March 2025. Informed consent from the church leadership and then from all participants prior to their interviews was sought. Each participant was informed of the research purpose, their role and their rights, which included the ability to withdraw from the research at any time without penalty. Informed consent was not only verbal but also documented to guarantee that all participants had a comprehensive understanding of the research objectives.

The potential emotional distress that participants could have experienced when discussing their personal experiences was considered. It was important that the participants felt safe and at ease during the entire process. They did not have any emotional distress, and interviews could all be conducted and completed. However, contingencies (such as referral to local, free counselling services) were in place to support participants if they had become distressed. The research was conducted with empathy and respect as a result of the contemplation of the emotional well-being of the participants.

## Results

Four themes emerged from the data analysis. Firstly, ‘Physical barriers to participation’ demonstrates the challenges that persons with disabilities encounter to accessing and participating in church activities in-person. Secondly, ‘Emotional and spiritual support’ forms a contrast with the barriers and frustrations described in theme 1 as participants describe the space of safety, support and spiritual upliftment which the online Bible study has provided. Thirdly, ‘Role of technology’ explores the benefits for access, as well as the frustrations, that technology holds. Finally, ‘Critical issues of voice, silence and exclusion’ considers the nuances of inclusion and exclusion as participants recognise the online space as valuable but still desire inclusion and a voice within the main church congregation.

### Physical barriers to participation

Participants described encountering barriers to participating in church services in-person, namely, mobility issues and difficulties in accessing transportation. The online Bible study group was formed to meet these challenges.

#### Mobility issues

Participants noted the struggle they face in attending in-person church services. They attribute this struggle to their impairments in combination with environments that do not accommodate their impairments. For instance, Kindness said: ‘Due to my mobility issues, I wasn’t able to attend church’. But later they added: ‘In our church building, there’s no accommodation made for persons with mobility disabilities’. Courage similarly shared:

‘OK. The one thing I would say is make it more accommodating, like in my case … I can hardly walk. I’ve got to make use of a crutch, or a Walker and it is so frustrating … When I can’t even go to church honest with you, I do not attend the church anymore … because of the stairs I cannot do the stairs.’ (Courage)

These issues could be avoided if universal design principles were applied, ensuring buildings are accessible to everyone (Carter 2023). These results align with the social model of disability, which asserts that disability stems from the social relationships that marginalise and disadvantage persons with impairments (Thomas 2004). According to Carter (2023), congregational attitudes shape the accessibility and inclusiveness of physical venues. If a congregation does not anticipate or expect persons with disabilities to actively participate, they may unintentionally exclude them by neglecting to provide the appropriate accommodations.

#### Transport difficulties

Access to transport is a widely cited barrier to participation for persons with disabilities, especially in low- and middle-income countries (Duri & Luke 2022; Maart et al. 2007; Vergunst et al. 2015). Beyond physical access challenges, participants mentioned issues of safety:

‘Traveling with public transport wasn’t safe or confident for me … I didn’t attend church for many months due to travel difficulties.’ (Peace)

Even where specialised transport systems exist, these frequently fail to satisfy user requirements, whether because of restricted availability, prolonged wait times or insufficient reliability. Dial-A-Ride, for example, is a specialist transportation service that offers door-to-door transit for persons with mobility and other impairments. Kindness said of this service: ‘Dial-a-Ride is a huge let down for anybody with mobility issues’. These barriers form a contrast with the ways in which participants describe the online Bible study group as a supportive and safe environment.

### Emotional and spiritual support

In contrast to the challenges, frustrations and insecurity described by participants in the previous section, they describe feeling encouraged, safe and valued within the online Bible study group. The support and care from fellow group members were often mentioned as creating a sense of belonging and community and spiritual well-being.

#### Supportive and safe environment

Participants describe the relationships in the group as reciprocal where they both give and receive support, fostering a sense of mutual care and belonging. Kindness said: ‘We do life together’. This highlights how the group functions as a close-knit community beyond just studying the Bible. The phrase ‘do life together’ implies a deeper and broader relationship. This group can be seen to play a key role in combating the isolation that characterises the experience of many persons with disabilities, due in large part to the inaccessibility of the physical environment that limits their social participation (Macdonald et al. 2018; Watermeyer & Swartz 2016).

Another positive aspect of the group is safety from the fear of being judged. Love described this as the freedom that members felt to ‘be themselves’. Other participants shared that the group made space for them to share difficult experiences while feeling safe:

‘Yes, we in this group … you’re safe to express how your family may have hurt you.’ (Peace)

‘There’s no fear of thinking, ‘What are they going to think of me?’ … We do not judge each other in this group. We are open. We are free-spirited. It is amazing.’ (Courage)

These experiences align with work that shows positive correlations between improved mental health and disability group membership (Zapata 2022). These experiences also demonstrate an important contrast with the ways in which persons with disabilities can often feel pressured to silence certain experiences in favour of portraying an image of capability or stoicism (Watermeyer 2009; Watermeyer & Botha 2023) or may feel prohibited from expressing dissatisfaction or complaint (Lourens 2018). This can be particularly so in faith-based spaces where persons with disabilities may feel that admitting struggle will be misconstrued as a failure of their faith (Botha in press).

#### Spiritual growth and guidance

Alongside the emotional support, participants describe the spiritual growth they experienced in the group, particularly how, through the group, the Holy Spirit had helped them to grow closer to God. In Christian faith, ‘The Holy Spirit’ is seen as a guide that plays a crucial part in a believer’s spiritual life by guiding them toward a fuller understanding and realisation of God’s truth, especially as revealed in the scriptures (Yong 2010). Peace mentioned how, in moments of personal anguish and prayer, ‘The more the Holy Spirit is leading me to scripture, and now I understand … I have an assignment from the Lord’. This corresponds with Jesus’ statement in John 14:26: ‘But the Advocate, the Holy Spirit, whom the Father will send in my name, will teach you all things and will remind you of everything I have said to you’ (NIV). In this scripture, the Holy Spirit presents as a guide to Christians in understanding their purpose, particularly when going through times of spiritual adversity.

Similarly, Courage talks about their experience of being taken to ‘the next level with the Lord’. This presents the Holy Spirit’s influence as reflected in Romans 8:26:

In the same way, the Spirit helps us in our weakness. We do not know what we ought to pray for, but the Spirit himself intercedes for us through wordless groans. (NIV)

This demonstrates that persons with disabilities may still have a fulfilling spiritual life even if they are unable to attend typical church services, fostering a sense of belonging and inclusion online (Carter 2024).

Another central aspect of the group’s gatherings is the provision of consistent spiritual encouragement. As Courage observes, ‘Every Wednesday morning, you feel so uplifted spiritually’. Peace added: ‘This group is so, so very important for me because we really encourage one another’. By giving God’s love, hope and guidance, the church can help people get to know each other and build relationships (Zondi 2020). The church pulls its members up through shared faith, fellowship and support, giving them the tools they need to build a strong, united base that can withstand hardship and promote peace and unity. Hutchings (2011) and Russell (2016) provide added evidence for this by underlining the fact that fellowship within religious communities plays a vital role in the transmission of religious identity and gives crucial support, particularly during times of difficulty. This positive influence is clear in participants’ experiences within the Bible study group.

### Role of technology

Technology takes centre stage in supporting connections and enabling participation in this setting. While there are clear advantages, participants’ accounts also reveal challenges with access to technology.

#### Accessibility through technology

Smartphones and other devices bridge the gap caused by physical barriers. Peace said: ‘If it wasn’t for smartphones, we would be disconnected from the world of information’. Similarly, Love shared: ‘With modern technology, we do video calls. It’s almost like we are right in each other’s company physically’. These participants describe engaging in fellowship in a way which suggests that this vital spiritual component need not be compromised in the online space (Hutchings 2011).

Technology also enables comfort and safety, which is particularly beneficial to persons with chronic health conditions or severe mobility impairments. Love shared: ‘Technology allows us to participate even from our beds’. Again, this forms a stark contrast with the struggles described in theme 1 and strengthens the view of this online group as a safe and non-judgemental space. This supports the notion that the online church holds potential to promote the spiritual and emotional well-being of persons with disabilities (Russell 2016).

#### Technology barriers

Although technology offers many benefits, barriers remain for persons with disabilities. Even when they have access to assistive devices, as Peace advises, ‘Love has to often help me and log me into the chat as I don’t always have help’. Technology can perpetuate exclusion. Online platforms are often inaccessible or not user-friendly, offering limited support for those who may live alone with no help or may not have an appropriate assistive device to access the platform (Smit 2021). Socio-economic constraints to accessing assistive technology are also a factor. Participants shared that they often needed support to engage with technology. Courage, for example, said: ‘Luckily, I’ve got my children around me, and they help me a lot’. Kindness described how they attempt to help other group members with the technology, ‘I provide step-by-step guides on using WhatsApp calls’.

These potential barriers and challenges must be taken into account when churches consider online alternatives as access measures. It is important to avoid the assumption that technology presents a seamless solution. Rather, the collaboration between the church and its members, which includes persons with disabilities, should be ongoing in looking for ways to be more inclusive (Amenyedzi 2024). The church should use the ability of both members and specialised organisations to make informed decisions about digital platforms and devices to use.

### Critical issues of voice, silence and exclusion

While participants were technically included in the online, disability accessible Bible study group, many found themselves having to advocate for their inclusion within the broader church community. The concerns of voice and silence are important to evaluate the true efficacy of inclusive measures. Alhuzail and Levinger (2022) note that ableist cultural norms not only silence the voices of persons with disabilities but also limit their visibility in public spaces, resulting in a form of enforced silence around their identities and needs. The Bible study group members expressed feeling that they had to ‘speak up’ for themselves in relation to the rest of the church congregation. Kindness said: ‘I make sure that the special needs group gets heard’. The sense here is that, if they did not make sure they were heard, they would be at risk of being forgotten.

Kindness also underscored the significance of accommodating persons with disabilities, emphasising that their meaningful engagement is dependent upon thoughtful modifications: ‘Our group would like to be involved, and of course, our involvement would require accommodation in order to help’. In this, the desire for involvement in the main church is clearly expressed. This prompts the question of whether a segregated group is truly an access and inclusion solution, or whether it might in fact perpetuate isolation, invisibility and silence.

On the contrary, Peace shared an experience of inclusion in the broader church congregation that was positive and affirming. Although initially anxious to participate in a church outreach, simple adjustments and care from the congregation made this a good experience:

‘The evangelism group? I’m like, wow, you can’t put me in the evangelism group. How am I going to get around? … People at my church said, “No, sister. You can. You can stand, you can take the mic, and you can bring hope to others.”’ (Peace)

Here, Peace is recognised as being able to make a contribution and as having a divine purpose. Simple adjustments and support can help persons with disabilities use their voices effectively in the church’s physical spaces, as shown by this case.

While some churches are actively working to eliminate barriers to full participation, others have not yet made accessibility a priority (Carter et al. 2023). The lived experiences of persons with disabilities can offer valuable insights on how churches can become more inclusive. Their recommendations can be an important factor for church leaders to ensure that the church environment is both physically and socially accommodating. Moreover, the tension between purported societal openness to inclusion of persons with disabilities and the persistence of exclusionary practices highlights the need for continued advocacy to ensure that inclusion is not merely a legal formality but a lived reality.

## Discussion

The research findings demonstrate the potential of online spaces to enable spiritual connection, growth and support for persons with disabilities. Participants identified the physical inaccessibility of traditional church structures as a significant challenge. These barriers demonstrate both the physical restrictions imposed by particular impairments and the larger cultural apathy towards providing accessible environments leading to disability (Shakespeare 2013). The lack of accessible design not only makes it impossible for persons with disabilities to get into the building but also supports social exclusion by making them feel like they don’t belong to the community and religious spaces (Watermeyer & Swartz 2016). These barriers are also indicative of the relationship of the church to disability as an expected and valued aspect of a diverse congregation (Carter et al. 2023). Reliance on inadequate specialists or public transport further separates persons with disabilities from access to spaces of spiritual development and well-being, meaning that efforts towards inclusivity must think beyond the bounds of the church building (Duri & Luke 2022; Maart et al. 2007; Vergunst et al. 2015).

In contrast to these challenges encountered in conventional church settings, participants describe receiving emotional and spiritual support in the online space. Participants frequently talked about the sense of community and encouragement they experienced within the group, characterising it as a ‘prayer answered’. Participants felt enabled to openly and without fear of judgement discuss personal challenges, family issues, pain and spiritual issues. This supportive environment is in stark contrast to the isolation that often characterises persons with disabilities’ experiences in societies where disability is frequently stigmatised and sharing personal struggles may be met with misunderstanding or silence (Lourens 2018; Watermeyer 2009; Watermeyer & Swartz 2016). This fear of being misunderstood may be exacerbated in religious settings because of beliefs such as an imperative for Christians to demonstrate long suffering with Christ (Botha in press). The participants’ accounts suggest that the Bible study group counteracted a societal pressure to behave with stoicism, enabling them to embrace and share vulnerability. Scholars have suggested that the ability to ‘be real’ about lived experiences, which may include struggle or pain, is essential to promote belonging and well-being for persons with disabilities (Lourens 2021; Watermeyer & Botha 2023; Watermeyer & Swartz 2008).

The spiritual lives of participants were also enriched through attending the online group. They described growing closer to God and receiving guidance from the Holy Spirit during difficult times, consistent with biblical teachings on the Holy Spirit’s role in guiding Christians towards spiritual awareness and resilience. Consistent spiritual encouragement emerged as one of the group’s primary benefits. This strengthens the members’ sense of purpose and allows them to keep a strong connection to their faith, supporting the assertion that persons with disabilities can experience profound spiritual joy outside traditional church settings (Carter 2024). According to Zondi (2020), the church’s role in showing God’s love and establishing fellowship is critical to develop unity and resilience, which is consistent with the experiences shared by group members.

Technology is key to enabling these emotional and spiritual benefits. That a participant described joining the group from her bed shows how technology can be enabling and speaks to the openness of the group to meet people where they are at. However, technology also introduces barriers, and hence, it should not be viewed as a seamless solution. Issues such as usability, affordability and accessible design, which have been identified as issues in promoting inclusive digital participation, resonate (Smit 2021).

Similarly, the online group in itself should not be viewed as a complete solution to the exclusion of persons with disabilities from conventional in-person gatherings of this congregation. Although members are welcome and supported in the online Bible study, which has been designed with inclusion as its aim, true inclusion in the larger church remains a challenge. This must encourage us to take a critical view of inclusive measures to question both their benefits and their limits, and perhaps even the ways in which they may re-inscribe segregation (Botha et al. 2023). In contrast, even minor changes may empower persons with disabilities to offer their voices in unexpected ways, as seen in the story shared by Peace. The Bible study participants’ experiences might give us a significant insight for church leaders looking to establish a more inclusive environment in which persons with disabilities are valued and actively encouraged to engage.

### Recommendations

Based on the results, the following recommendations are made:

- *Improving accessibility*: Faith-based organisations should consider creating accessible online platforms for remote engagement with church activities, but not view these as a complete solution to disability inclusion in their congregations.
- *Training for leaders*: Church and other faith leaders should be equipped with knowledge on disabling barriers, the design of inclusive spaces and the ways in which religious discourses can negatively impact on inclusion of persons with disabilities in church and faith-based organisations.
- *Fostering community and belonging*: Churches and other faith-based organisations should develop intentional programmes to build communities that recognise and include disability as diversity. This could involve mentorship programmes, integrated social events and consulting with members with disabilities on ways to be more inclusive.
- *Ongoing research and evaluation*: Establishing a feedback loop will assist faith-based organisations to develop inclusive planning and programming.
- *Collaboration with disability advocacy groups*: Faith-based organisations should form collaborations with disability advocacy groups as information resources on assistive technology, digital inclusion and inclusive design of physical spaces and programmes.
- *Incorporating diverse perspectives*: Involve persons with disabilities in decision-making, planning and leadership structures of the church or other faith-based organisations to ensure that their needs and perspectives are represented.

### Limitations

We acknowledge the limits of this research as a small qualitative study. Although we cannot make generalisable claims on experiences of persons with disabilities in accessing spiritual spaces, we believe that the perspectives of the participants in this study offer some insight into the complexities of inclusion and exclusion, and the potential and challenges of the digital realm for fostering spiritual connection, growth and well-being in the lives of persons with disabilities.

## Conclusion

This article presented results from a study that investigated the experiences of persons with disabilities participating in an online Bible study group hosted by a Pentecostal Christian church in Cape Town, South Africa. The study found that the group promotes spiritual growth, provides a secure environment and uses technology to meet accessibility needs. However, societal barriers still present challenges to full participation and inclusion in the community of the larger church congregation. The digital realm offers only partial inclusion and may not adequately provide persons with disabilities with the sense that they are welcome and that they belong within the church community.

This research illustrates the complexity surrounding the experiences of persons with disabilities in faith-based environments. The stories that participants have revealed highlight the need for a change in viewpoint inside the church community, one that stresses acceptance, support and empowerment, rather than only healing or normalising. Church communities need to anticipate that persons with disabilities will form part of their congregations and make efforts to review their practices and programming with universal access and inclusive care in mind. Here, care is understood not as a help or medical intervention, it should underpin a commitment to design environments and build relationships that remove participation barriers and promote equity (Evans, Hsu & Boerma 2013).

This is particularly needed as engagement in faith activities holds significant benefits for persons with disabilities, providing hope, purpose and a secure sense of identity. In the end, the demand for inclusion reflects the conviction that every person is formed in the image of God and is deserving of love and acceptance. Universal inclusion complements the essential principles of Christianity and should therefore not be seen as a purely legal requirement within church communities.
